# Massive Cervicothoracic Subcutaneous Emphysema and Pneumomediastinum Developing during a Dental Hygiene Procedure

**DOI:** 10.1155/2017/7016467

**Published:** 2017-04-13

**Authors:** Gabriele Bocchialini, Serena Ambrosi, Andrea Castellani

**Affiliations:** Maxillo-Facial Surgery Unit, Spedali Civili di Brescia, Piazzale Spedali Civili 1, 25123 Brescia, Italy

## Abstract

Subcutaneous emphysema is rare during or after dental procedures (usually extractions). Here, we describe the case of a 65-year-old woman who developed massive cervicothoracic subcutaneous emphysema and pneumomediastinum during a dental hygiene procedure employing an artificial airflow. She was diagnosed based on clinical manifestations and computed tomography (CT). CT revealed massive subcutaneous emphysema extending from the superior left eyelid to the diaphragm. We describe the clinical and radiological characteristics of this rare case.

## 1. Introduction

A variety of procedures can cause subcutaneous emphysema and pneumomediastinum, including injury, head and neck surgery, mechanical ventilation, and invasive procedures such as bronchoscopy. A few cases have been observed during dental procedures, principally third molar extraction [1, 2]. Emphysema can also be caused by an increase in mouth air pressure caused by playing a wind instrument [3] or blowing up a balloon [4]. Emphysema developing during or after dental procedures is rare; most cases have been limited to the head and neck, with only a few involving the mediastinum [5].

We present a rare case of a patient diagnosed with massive subcutaneous emphysema extending from the superior left eyelid to the diaphragm. The condition developed during a mandibular dental hygiene procedure in the absence of any visible intraoral incision.

## 2. Case Report

A 65-year-old woman was referred by her dentist to the emergency department of Spedali Civili Brescia, Italy, with a large swelling of the face and the neck that commenced at the upper left eyelid. Pain was not aggravated on palpation, but dysphagia, dyslalia, and subcutaneous crepitus were evident. An intraoral examination revealed no visible incision; slight bleeding was apparent around an implant in the region of 34. She reported that she had undergone an airflow procedure performed by a dental hygienist in the region of the implant earlier the same day and had felt pain in the left submandibular region during the procedure. She was diagnosed with subcutaneous emphysema (procedural complication).

Her vital signs were as follows: heart rate 65 beats per minute; blood pressure 145/90 mmHg; respiration 19 breaths per minute; and oxygen saturation 96%.

To evaluate the dysphagia and dyslalia, we obtained CT from the maxillofacial region to the thorax. These revealed significant soft-tissue emphysema extending from the left parietal region to the left soft tissue of the face, then bilaterally to the paraspinal muscles of the neck and the pterygoid regions, and posteriorly to the pharynx.

More distally, the emphysema splayed the vascular bundle and the thyroid lobes, widened the pectoral muscles posterior to the clavicles, descended to the mediastinum (where it was evident principally in the anterior part of the perivascular adiposity), and surrounded the trachea and oesophagus posteriorly.

**Figure 1:**
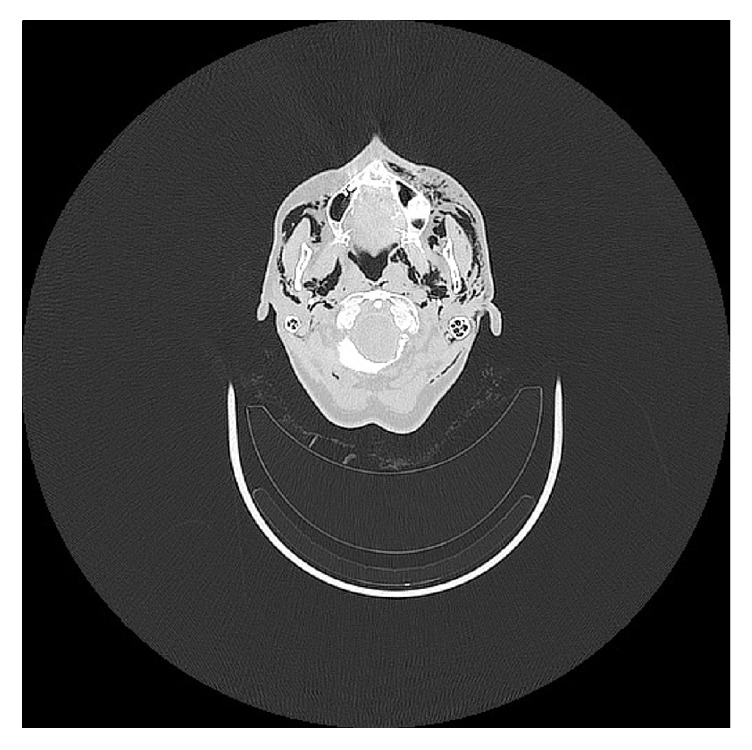
Computed tomographic axial view of the emphysema in the maxillary region.

**Figure 2:**
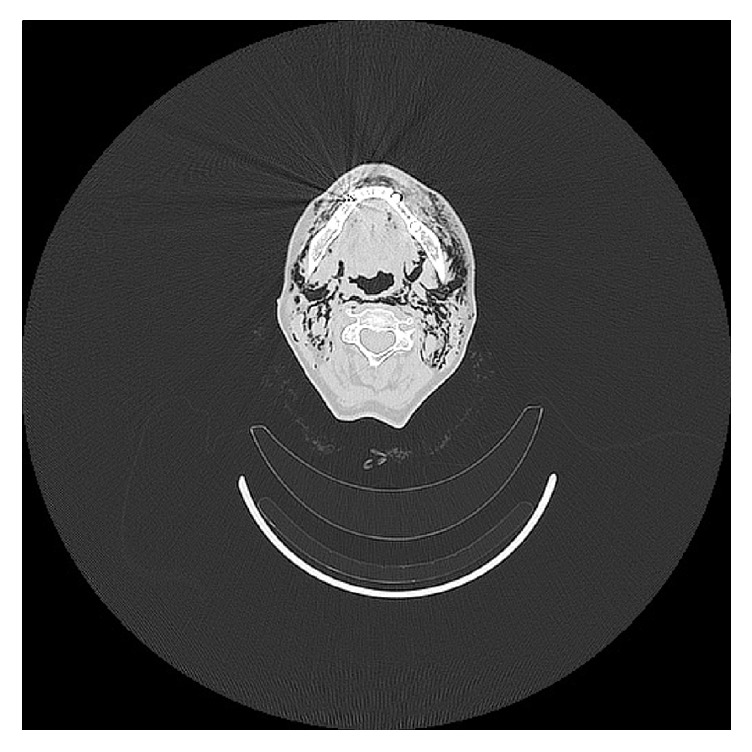
Computed tomographic axial view of the emphysema in the mandibular region.

**Figure 3:**
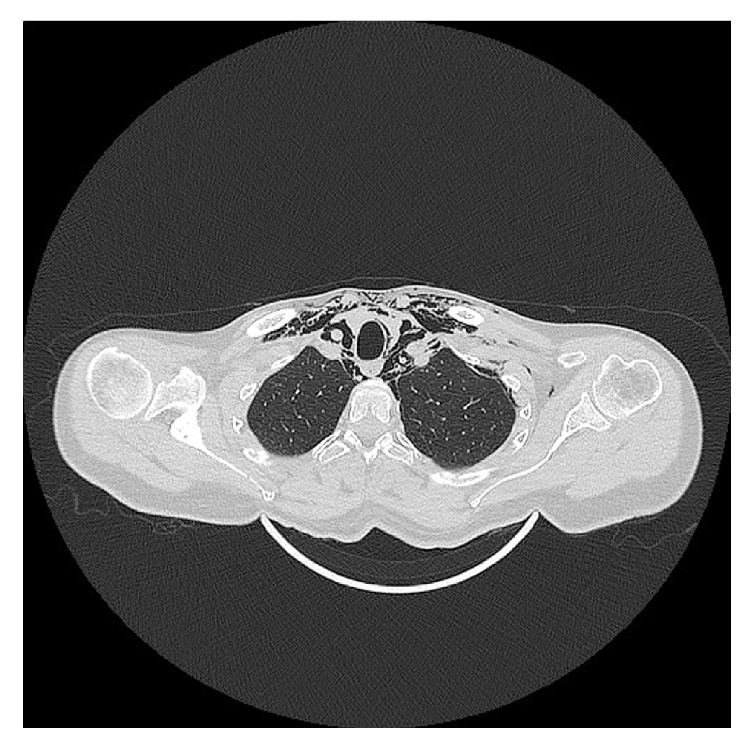
Computed tomographic axial view of the emphysema in the upper thorax, splaying the vascular bundle and running posterior to the clavicles with widening of the pectoral muscles.

**Figure 4:**
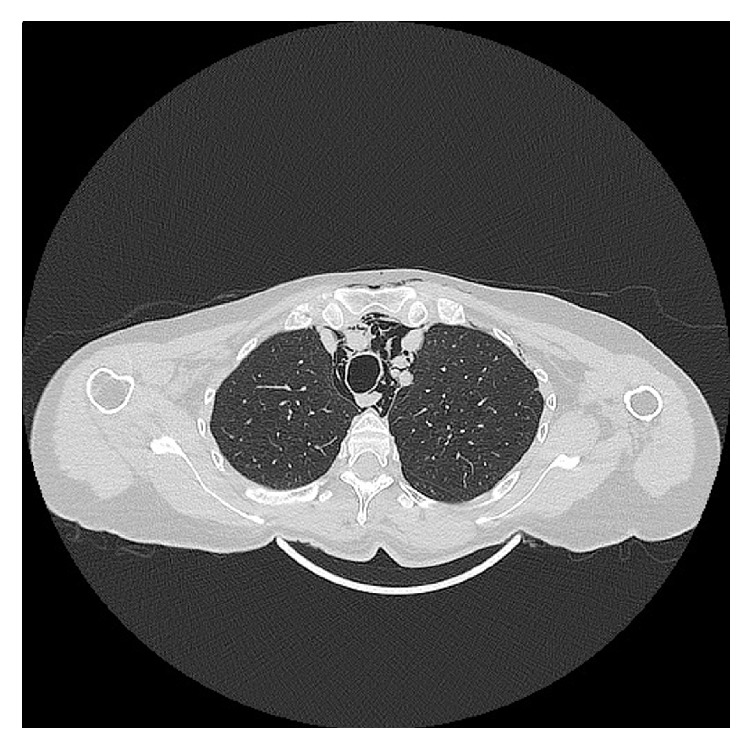
Computed tomographic axial view of the emphysema descending to the mediastinum, where it is evident mainly in the anterior region of perivascular adiposity and surrounding the trachea and oesophagus posteriorly.

The emphysema terminated in the region of the upper diaphragm (Figures 1–4).

To prevent the expansion of the emphysema, the patient was immediately hospitalised in the Maxillofacial Surgery Unit and was prescribed intravenous antibiotics because of the high risk of infection associated with the access of large amounts of air and water to soft tissue during a dental procedure. Indeed, dental compressed air, and not sterile water, contains* Legionella* and* Pseudomonas*, rendering antibiotic therapy and microbiological monitoring even more critical [6–9].

The patient was discharged 4 days later.

## 3. Discussion

Turnbull, in 1900, was the first to describe subcutaneous emphysema and pneumomediastinum developing after dental treatment, when a musician blew a bugle immediately after tooth extraction [3]. Heyman and Babayof reviewed 75 cases of emphysema developing after dental treatment from 1960 to 1993 [10], and Arai et al. presented another 47 cases from 1994 to 2008 [5].

A variety of procedures can cause subcutaneous emphysema and pneumomediastinum, including injury, head and neck surgery, mechanical ventilation, and invasive procedures such as bronchoscopy.

A few cases have been observed during dental procedures, principally third molar extraction using an air turbine hand piece [1, 2]. Some cases have been associated with the use of a dental laser, including CO_2_, Nd:YAG, and Er:YAG lasers. Fewer cases have been described after restorative or periodontal treatment that required no mucosal incision using peroxide hydrogen or sodium hypochlorite irrigants [5].

Swelling, dysphagia, chest pain, and crepitus are common signs and symptoms of emphysema and may develop immediately or within a few hours or days of the triggering procedure [1, 11]. Features suggestive of pneumomediastinum are dyspnoea with a brassy voice, chest or back pain, or the Hamman sign [5, 12]. The differential diagnosis of emphysematous complaints includes allergic reactions, haematoma, cellulitis, and angioedema [13]. When the diagnosis is difficult, the best option is empirical treatment as for an anaphylactic reaction until a definitive diagnosis is possible [7]. Emphysema is sometimes detected the day after a procedure [14].

Most patients who develop emphysema after dental procedures exhibit local symptoms that are benign and self-limiting in the clinic. Complications include the need for tracheostomy or thoracic drainage, mediastinitis, an air embolism [2, 7, 11, 15, 16], pneumoperitoneum, pneumopericardium [17], and necrotising fasciitis [18]. Therefore, emphysema must be distinguished from gasses released by necrotising fasciitis with the help of serial CT imaging when necessary [7]. CT is the most useful imaging technique, affording excellent detail [5].

Although infection is not usually observed in subcutaneous emphysema, this condition has developed in some cases. The use of a prophylactic antibiotic therapy is recommended because the introduction of air, and not sterile water [9], and the migration of oral cavity microorganisms to the mediastinum [19] could have serious effects on the patient's health. For these reasons, simple bed rest with antibiotics has always been the therapy of choice [20, 21].

However, there have been reports of death due to complications such as mediastinitis, pneumothorax, cardiac tamponade, cardiac failure, and air embolism [21, 22].

## 4. Conclusion

Our case is unusual in terms of the extent of emphysema noted and the simple dental procedure that triggered the problem. We present this case to emphasise that no matter how simple the planned procedure is, something can always go catastrophically wrong.
